# Effects of Tongue-Strengthening Self-Exercises in Healthy Older Adults: A Non-Randomized Controlled Trial

**DOI:** 10.1007/s00455-020-10216-w

**Published:** 2020-11-19

**Authors:** Jitsuro Yano, Shinsuke Nagami, Tomonori Yokoyama, Katsuya Nakamura, Miyu Kobayashi, Yuki Odan, Miyako Hikasa, Kozo Hanayama, Shinya Fukunaga

**Affiliations:** 1 Department of Speech-Language Pathology and Audiology, Faculty of Rehabilitation, Kawasaki University of Medical Welfare, 288 Matsushima, Kurashiki, Okayama 701-0193 Japan; 2 Division of Speech-Language-Hearing Therapy, Rehabilitation Center, Kawasaki Medical School Hospital, 577 Matsushima, Kurashiki, Okayama 701-0192 Japan; 3 Department of Occlusal and Oral Functional Rehabilitation, Graduate School of Medicine, Dentistry and Pharmaceutical Sciences, Okayama University, 2-5-1 Shikata-cho, Kita-ku, Okayama, 700-8525 Japan; 4 Division of Speech-Language-Hearing Therapy, Rehabilitation Center, Kawasaki Medical School General Medical Center, 2-6-1 Nakasange, Kita-ku, Okayama 700-8505 Japan; 5 Department of Sensory Science, Graduate School of Health Science and Technology, Kawasaki University of Medical Welfare, 288 Matsushima, Kurashiki, Okayama 701-0193 Japan; 6 Department of Preventive Rehabilitation, Kurashiki Heisei Hospital, 4-4-7 Oimatsu-cho, Kurashiki, Okayama 710-0826 Japan; 7 Department of Day Care Rehabilitation, Kurashiki Geriatric Health Services Facilities, 4-4-7 Oimatsu-cho, Kurashiki, Okayama 710-0826 Japan; 8 Department of Rehabilitation Medicine, Kawasaki Medical School, 577 Matsushima, Kurashiki, Okayama 701-0192 Japan

**Keywords:** Tongue strength, Tongue pressure, Home-based exercise, Self-exercise, Swallow, Dysphagia

## Abstract

Tongue-strengthening exercises (TSE) using a device have been proposed as an intervention for improving tongue strength and endurance. However, devices for TSE have been expensive and difficult to manipulate and are not commonly used in home or clinical settings. This study therefore aimed to investigate whether tongue-strengthening self-exercises (TSsE) using a tongue-strengthening self-exercise tool at home can improve tongue strength in healthy older adults. This study included 27 participants (exercise group, *η* = 16, 7 men, 9 women, median age 84.5 years; control group, *n* = 11, 2 men, 9 women, median age 79.0 years). Exercises in the exercise group consisted of pushing the anterior tongue against the hard palate 30 times, 3 times a day, 5 days a week, for 8 weeks using a tongue-strengthening self-exercise tool. This tool is available in five levels of hardness. The most suitable hardness of the tool for each participant was calculated based on 60% of maximum tongue pressure (MTP) during the first 2 weeks of the training period and 80% of MTP for the remainder of the training period, as assessed using a tongue pressure-measuring device. The exercise group showed a significant improvement of 4.1 kPa in MTP (an 11.53% increase) and 4.53 s in endurance of tongue pressure (ETP) (a 99.86% increase). Furthermore, adherence in the exercise group was 99.2%. In conclusion, performing TSsE for 8 weeks was effective for increasing MTP and ETP in healthy older adults. This indicates that TSsE may be useful in older individuals at home to prevent age-related tongue muscle weakness.

## Introduction

The prevalence of frailty increases with age and the associated risk of health problems such as institutionalization, hospitalization, and falls, and death is recognized internationally [1, 2]. Previous studies have linked tongue strength and frailty, and a decrease in tongue strength is known to affect feeding, swallowing functions, and nutrition [3]. In addition, the tongue plays an important role in swallowing, and weakening of the tongue muscles is one of the most common age-related changes in older individuals [4]. It is therefore important for older adults to maintain and improve tongue strength in order to slow the onset of frailty and maintain a healthy lifestyle. To prevent and improve age-related muscle weakness, resistance training is an intervention frequently used for older adults [5, 6].

Previous studies have shown that tongue-strengthening exercises (TSE) increase tongue pressure in stroke patients, patients with acquired brain injury, and healthy participants using a tongue-strengthening measuring device [7, 8]. Tongue pressure was the one indicator used to evaluate motor function of the tongue, as the force of the tongue press against the palate. In our previous studies, TSE using the device increased not only tongue pressure, but also the area of the geniohyoid muscle, which plays crucial roles in the swallowing mechanism of healthy young adults [9, 10]. These effects of TSE were maintained without any further exercise in the subsequent 8-week detraining period [11–14]. Older adults should continue as long as possible with these exercises to prevent age-related tongue muscle weakness [15, 16]. However, devices used to perform TSE have remained expensive and difficult to manipulate and thus are not commonly used in the home or clinical settings without supervision by a therapist. A home-based TSE program with easy-to-perform exercises should thus be developed for older adults.

When older adults perform home-based exercises, adherence is crucial, and evidence suggests that older adults who keep to the prescribed exercise schedule improve swallowing outcomes [17, 18]. However, the adherence of older adults to home-based exercises declines over time [19, 20]. This reduction in adherence in older adults appears to be associated with psychological factors such as cognitive ability, stress, and depression [20, 21]. Older adults with a background of cognitive decline may not adapt to a tongue measurement device that is difficult to manipulate, which can lead to poor adherence. A simple, straightforward methodology is thus key to the design of home-based programs to increase adherence among older adults [22, 23]. However, direct evidence for the utility of TSE in the home by the individual remains lacking.

In this study, we aimed to investigate whether tongue-strengthening self-exercises (TSsE) using a tongue-strengthening self-exercise tool that is commercially available, inexpensive, and easy to use at home can improve tongue strength in healthy older adults.

## Methods

### Study Design and Participants

This was a controlled, non-randomized trial designed to investigate whether TSsE using a tongue-strengthening self-exercise tool can improve tongue strength in healthy older adults.

The study was undertaken at Kurashiki Heisei Hospital. Thirty-eight participants (exercise group, *n* = 24; control group, *n* = 14) who used day services for community-dwelling older adults were enrolled in this study. Recruitment to the exercise group was conducted between October 2019 and December 2019. Recruitment to the control group was conducted between November 2019 and January 2020. Informed consent was obtained from all participants before participation in this study. Eligibility criteria included age ≥ 65 years, no history of illnesses causing dysphagia (such as cerebrovascular disease or neuromuscular disease), the ability to eat regular food, and no complaints of swallowing problems such as coughing or choking during eating. Exclusion criteria were as follows: (i) disturbances of deglutition (Eating Assessment Tool-10 (EAT-10) score ≥ 3) [24, 25]; (ii) history of major surgery to the head or neck, or oral disease (other than routine tonsillectomy or previous tracheostomy); (iii) history of neurologic impairment (for example, Parkinson’s disease or multiple sclerosis); or (iv) inability to obtain informed consent because of cognitive impairment.

**Fig. 1:**
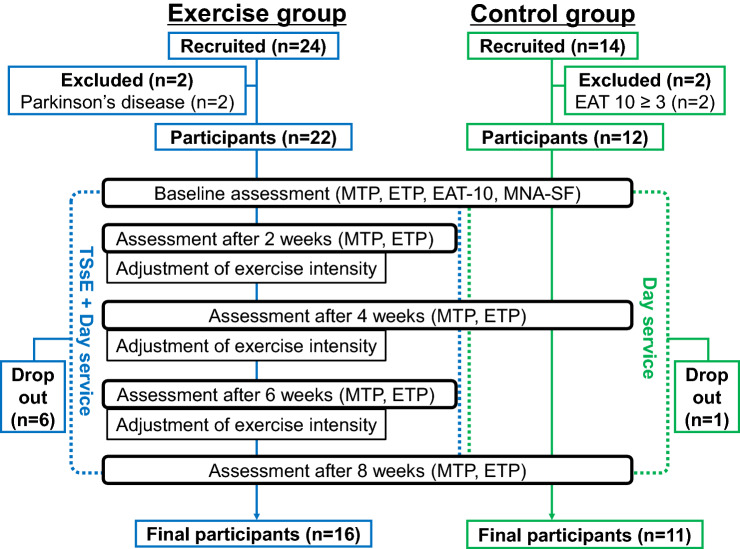
Study protocol for exercise group and control groups. *MTP* maximum tongue pressure, *ETP* endurance of tongue pressure, *EAT-10* Eating Assessment Tool-10, *MNA-SF* Mini Nutritional Assessment-Short Form

A flow chart describing participant flow through the study is shown in Fig. 1. Of the 38 participants enrolled in this study, 4 failed to meet the specified eligibility criteria. Of these, two potential participants in the exercise group were excluded due to neurologic impairment (Parkinson’s disease) and two potential participants in the control group were excluded due to disturbances of deglutition (EAT-10 scores 14 and 4, respectively). As a result, the exercise group comprised 22 participants and the control group included 12 participants. In the exercise group, 6 participants dropped out during the 8-week follow-up period because of illness (*n* = 2), scheduling mistakes (*n* = 3), or loss of motivation (*n* = 2). In the control group, 1 participant dropped out during the 8-week follow-up period because of a loss of motivation.

At the end of the study, complete data were available for 16 participants in the exercise group (7 men, 9 women; median age, 84.5 years; interquartile range (IQR), 75.25–87.75 years) and 11 participants in the control group (2 men, 9 women; median age, 79.0 years; IQR, 72.0–82.0 years). All study protocols were approved by the ethics committees of both Kawasaki University of Medical Welfare (Approval No. 18-054) and Kurashiki Heisei Hospital (approval no. H30-029).

### Equipment for Training and Measurement

#### Tongue Pressure Measurement Device

**Fig. 2:**
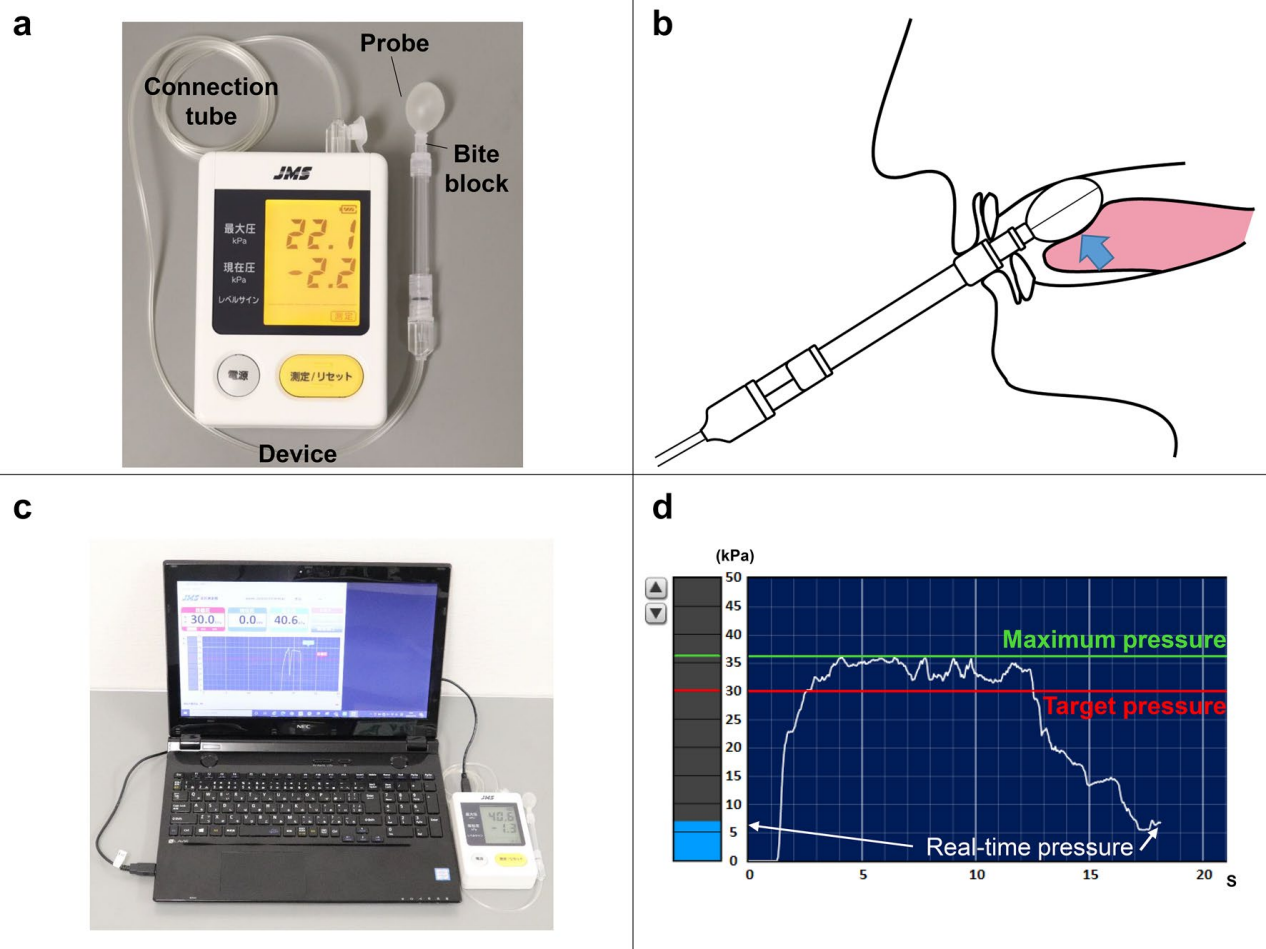
Tongue pressure measurement device. **a** Measurement device; **b** measurement setting; **c** measuring system; **d** PC display

A tongue pressure measurement device (TPM-01; JMS Co., Hiroshima, Japan) was used for the 8-week training program. Figure 2a shows the device, probe, and connecting tube. This balloon-type probe was inflated with air at an initial pressure of 19.6 kPa by turning on the pressurization switch. The balloon was approximately 18 mm in diameter, with a volume of 3.7 mL. This pressure was taken as the zero calibration. The participant was asked to hold the bite block so that the balloon could be placed between the tongue and anterior palate (Fig. 2b). Pressure measured by the device was transmitted in real time to a personal computer (Fig. 2c), where both current and maximal pressure values were displayed (Fig. 2d) and saved to a CSV file at 20 Hz. A new probe was used for every participant, both because of hygienic concerns and to minimize measurement errors due to possible variations in compliance of the bulb after extended use.

### Tongue-Strengthening Self-Exercise Tool

**Fig. 3:**
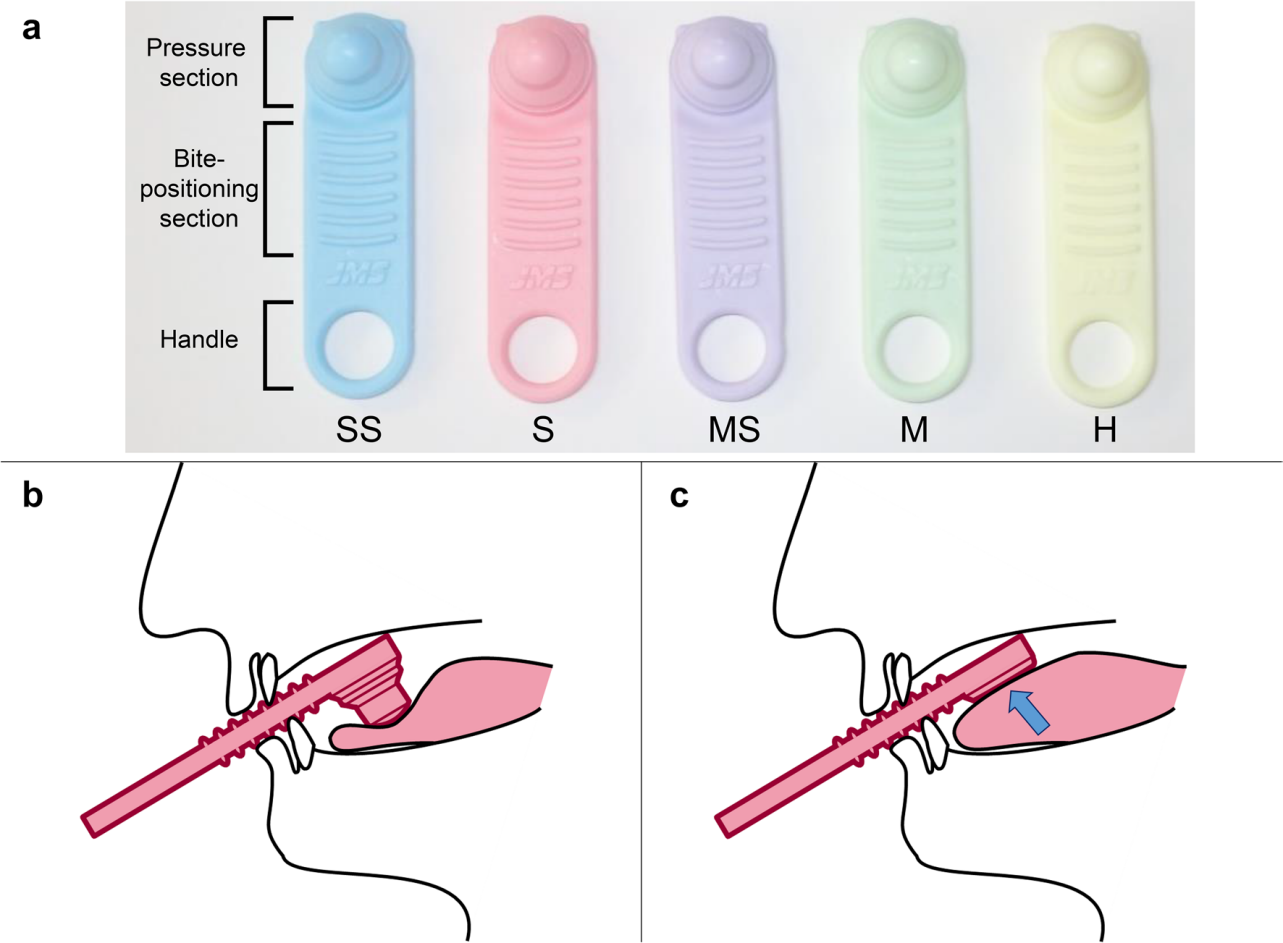
Tongue-strengthening self-exercise tool. **a** Level of hardness; **b** stable position; **c** pushing position. *SS* super-soft, *S* soft, *MS* medium-soft, *M* medium, *H* hard

All participants in the exercise group performed TSsE for 8 weeks using a tongue-strengthening self-exercise tool (Peco Panda®; JMS Co.) (Fig. 3). The tongue-strengthening self-exercise tool was made of thermoplastic styrenic elastomer. This tongue-strengthening self-exercise tool is available in five levels of hardness (super-soft, SS; soft, S; medium-soft, MS; medium, M; hard, H), selected according to the ability of the participant (Fig. 3a). The color and load of the press are set for each type (SS: blue, 5 kPa; S: pink, 10 kPa; MS: purple, 15 kPa; M: green, 20 kPa; H: yellow, 30 kPa). The tool comprises a pressure section, a bite-positioning section and a handle. The participant holds the handle to place the tool between the hard palate and tongue, ensuring stable positioning by biting on the bite-positioning section of the tool (Fig. 3b). The participant pushes the tongue upward against the tool until an indentation is made in the pressure section (Fig. 3c). Before the start of training, the participant pushed the pressure section of the tool with a finger outside the oral cavity to make sure it was depressed, then pushed the pressure section of the tool with the tongue in the oral cavity to ensure a similar dent was created.

### Method of Training

All subjects in the exercise group performed an 8-week training program involving TSsE as described by Robbins et al. [26], using the tongue-strengthening self-exercise tool at home (Fig. 1). Exercises in the exercise group consisted of pushing the anterior tongue against the hard palate 30 times as 1 set, performing 3 sets a day, 5 days a week, for 8 weeks using the tongue-strengthening self-exercise tool. Before starting the training program and at the start of the 2nd, 4th and 6th weeks of training, maximum tongue pressure (MTP) in each subject was measured using the JMS device to determine the target exercise intensity in the training. This target was calculated based on 60% of MTP during the first 2 weeks of the training period, then 80% of MTP for the remaining training period. The type of tongue-strengthening self-exercise tool for each participant was selected based on the target intensity. Relationships between type of tongue-strengthening self-exercise tool and target intensity were as follows: SS, target ≥ 5 kPa but < 10 kPa; S, target ≥ 10 kPa but < 15 kPa; MS, target ≥ 15 kPa but < 20 kPa; M, target ≥ 20 kPa but < 30 kPa; and H, target ≥ 30 kPa. Baseline and every 2 weeks were determined for assessment in the day service and were supervised by one of the researchers. Participants then received the tongue-strengthening self-exercise tool appropriate for the target intensity as their MTP changed.

Both the exercise group and the control group received day services for community-dwelling individuals. In addition, the facility provides outpatient rehabilitation services performed by physical, occupational, and/or speech-language-hearing therapists. These day services include physical exercises, cognitive training, and nutritional management. No participants in either group had performed resistance exercises for tongue muscles prior to this study.

### Outcome Measures

#### MTP

MTP was measured using the JMS device. Each subject was instructed to press the anterior tongue against the probe of the JMS device as hard as possible. The MTP selected for analysis was the maximum value from three trials for each subject.

MTP values taken at baseline and the start of the 4th and 8th weeks of the training program were used to evaluate training effects in each group, while values at baseline and at the start of the 2nd, 4th, and 6th weeks of the training program were used to determine the target exercise intensity in the training (Fig. 1).

The difference and rate of change in MTP between pre- and post-training program were calculated as [post-MTP − pre-MTP] and [((post-MTP − pre-MTP)/pre-MTP) × 100], respectively.

### Endurance of Tongue Pressure (ETP)

ETP values taken at baseline and at the start of the 4th and 8th weeks of the training program were used to evaluate training effects in each group.

ETP values were gathered following MTP measurement, after a break of at least 5 min. Participants were asked to sustain 50% of their MTP for as long as possible. Participants were always able to monitor their tongue pressure via the display of the personal computer, which showed the pressure relative to 50% of MTP in real time (Fig. 2d). Measurement of ETP was performed according to the method described by Solomon et al. [27]. Timing started when pressure met or exceeded 50% of MTP and stopped when the pressure dropped steeply and was either maintained at 40–50% of MTP for ≥ 2 s, or stayed < 40% of MTP for ≥ 0.5 s. Only one trial to determine ETP was performed for each participant because of the fatigue induced by the procedure. ETP was analyzed according to this rule on a personal computer from the CSV file. ETP was measured at the same time points as MTP.

Differences and rates of change in ETP between pre- and post-training program were calculated as [post-ETP − pre-ETP] and [((post-ETP − pre-ETP)/pre-ETP) × 100], respectively.

### Adherence

Adherence to TSsE was self-reported by participants in the exercise group. Participants were asked to document on a daily basis whether TSsE had been performed. Adherence was evaluated in each set as follows: 1 point = performed; 0 points = not performed. If the participant performed the training properly, adherence score would be 3 points per day, resulting in 15 points a week and 120 points in the 8-week study. The subsequent percentage adherence was calculated as the total sum of adherence scores, divided by 120 as the maximum point, then multiplied by 100.

### Statistical Analysis

A comparison of variables between the exercise and control groups at baseline were performed using Fisher’s exact test for categorical variables and the Mann–Whitney U test for continuous variables. Differences in MTP and ETP between baseline and the start of the 4th and 8th weeks of the training program in the same group were examined using two-way analysis of variance (ANOVA) (time [baseline, 4 weeks, 8 weeks] × group [exercise group, control group]) with post hoc Bonferroni correction, and effects sizes are reported as partial eta-squared values (*η*_p_^2^). The relationship between range of change (MTP and ETP) and adherence was analyzed using Spearman’s rank correlation coefficient. Statistical analysis was performed using IBM SPSS Statistics version 23 (IBM Japan, Tokyo, Japan), with significance set at *P* < 0.05.

## Results

### Participant Characteristics

Characteristics of the exercise and control groups at baseline are shown in Table 1. No significant differences in age, sex, weight, height, body mass index (BMI), EAT-10 score, Mini Nutritional Assessment-Short Form (MNA-SF) score [28], MTP or ETP were seen between groups at baseline.

### MTP

Two-way ANOVA (time × group) revealed a significant main effect of time (F_1.340, 29.485_ = 5.598, *P* = 0.017, *η*_p_^2^ = 0.203). Moreover, the main effect of group (*F*_1,22_ = 0.848, *P* = 0.367, *η*_p_^2^ = 0.037) was not significant, nor was the time × group interaction (*F*_1.340,29.485_ = 0.627, *P* = 0.480, *η*_p_^2^ = 0.028).

MTP increased significantly after the 8-week training program compared to before the program in the exercise group (*P* < 0.05). In the control group, MTP did not increase significantly after the 8-week training program compared to before the program (*P* = 0.861) (Table 2).

The difference and rate of change in MTP between pre- and post-training program did not differ significantly between exercise and control groups (difference, *P* = 0.148; rate of change, *P* = 0.195) (Table 3).

### ETP

Two-way ANOVA (time × group) revealed a significant main effect of time (*F*_2,44_ = 3.736, *P* = 0.032, *η*_p_^2^ = 0.145). Moreover, the main effect of group (*F*_1,22_ = 0.310, *P* = 0.583, *η*_p_^2^ = 0.014) was not significant, nor was the time × group interaction (*F*_2,44_ = 2.088, *P* = 0.136, *η*_p_^2^ = 0.087).

ETP was significantly increased after the 8-week training program compared to baseline in the exercise group (*P* < 0.01). In the control group, ETP did not increase significantly after the 8-week training program compared to before the program (*P* = 1.000) (Table 2).

The difference and rate of change in ETP between pre- and post-training program did no differ significantly between exercise and control groups (difference, *P* = 0.099; rate of change, *P* = 0.507) (Table 3).

### Adherence

**Fig. 4:**
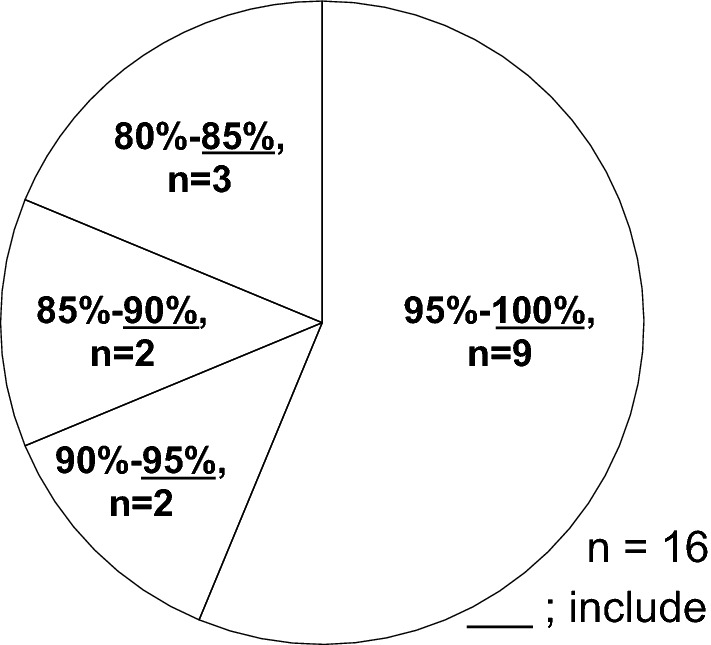
Adherence

**Fig. 5:**
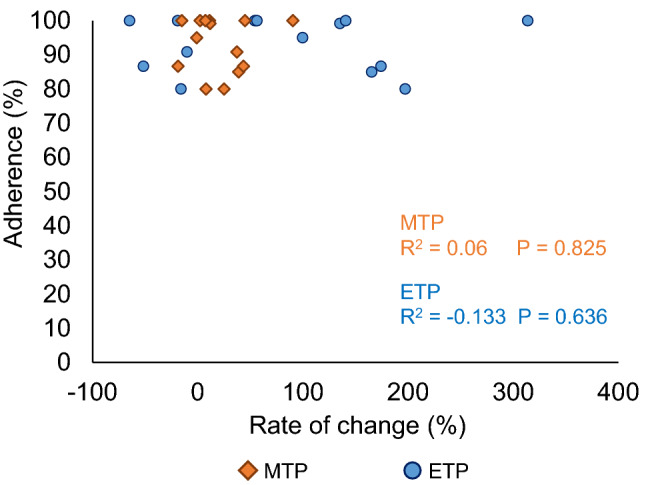
Relationship between adherence and rates of change in MTP and ETP in the exercise group. *MTP* maximum tongue pressure, *ETP* endurance of tongue pressure

Median adherence in the exercise group was 99.2% (IQR 86.7–100%). Adherence was > 95% but ≤ 100% in 9 subjects, > 90% but ≤ 95% in 2 subjects, > 85% but ≤ 90% in 2 subjects, and > 80% but ≤ 85% in 3 subjects (Fig. 4). This indicated that most participants in the 8-week trial had performed the training properly. Adherence did not correlate with rate of change between pre- and post-training program in MTP or ETP (MTP, *R*^2^ = 0.06, *P* = 0.825; ETP, *R*^2^ = − 0.133, *P* = 0.636) (Fig. 5).

## Discussion

This study showed that MTP and ETP significantly increased in healthy older adults after the 8 weeks of TSsE using the tongue-strengthening self-exercise tool at home. Previous studies have shown that TSE increased the MTP in stroke patients, patients with acquired brain injury, and healthy participants [7, 8]. In particular, many studies have examined TSE using devices and substantial evidence of its efficacy has been accumulated. However, devices used to perform TSE have remained expensive and difficult to manipulate. TSE using a device is thus not easy to perform and remains unsuitable for home-based programs. TSsE using a tongue-strengthening self-exercise tool that is commercially available, inexpensive and easy to use can be performed individually at home without the supervision of a therapist. However, few reports have described the use of this tool and training methods using this tool have not been established [29]. To the best of our knowledge, this represents the first study to investigate a quantitative self-training protocol for tongue muscles for use in the home. The present study yielded two important findings in testing the effectiveness of tongue self-training. First, TSsE were useful to increase both MTP and ETP in healthy older adults. Second, the home-based TSsE program maintained high adherence rates. Given these findings, TSsE appear as effective as TSE and are easily adaptable to various subjects.

The pre- and post-training comparisons of the exercise group showed a significant improvement of 4.1 kPa in MTP (an 11.53% increase) and 4.53 s in ETP (a 99.86% increase). Previous studies have already shown that TSE with this device increases MTP [7, 8]. Previous studies in healthy older adults using a tongue strength training device have shown improvements in MTP from 18 to 72% [11–13, 25, 30]. These values vary, but the effects of TSE using the device were expected to be greater than the effects of TSsE using the tongue-strengthening self-exercise tool in this study. The reason for this difference could be that the tongue-strengthening self-exercise tool for TSsE had only five training load settings, while the device for TSE provided a detailed load setting for each individual. During TSE, current and maximal pressure values were displayed digitally on the screen of the device in real time. This visual biofeedback might have affected the training effects of TSE. Previous studies have shown associations between visual biofeedback and tongue pressure [31–33]. TSE using the device might thus be more effective than TSsE using the tongue-strengthening self-exercise tool for healthy older individuals who want to increase MTP. However, the TSsE was thus considered very effective for home-based exercises. We should optimally be able to use both devices depending on the situation of the individual. Previous studies have reported that MTP declined with age, whereas ETP did not [15, 31, 34]. However, ETP decreased after meals in healthy adults [35] and with diseases such as Parkinson's disease [27] and amyotrophic lateral sclerosis [36]. Decreases in ETP have been suggested to affect the safety of swallowing [36]. Previous studies of TSE have rarely used ETP as an outcome. Clark et al. showed that ETP significantly increased by TSE using a device [37], and our findings supported their results. Besides, the declines in MTP seen with age not only increase the risk of aspiration, but also prolong mealtimes [38, 39]. Since post-meal fatigue decreases ETP [35], a prolonged mealtime might further decrease ETP [34, 40]. The TSsE in this study may thus be effective for increasing ETP and improving the safety of swallowing during extended mealtimes.

In this study, adherence rates (99.2%; IQR: 86.7–100%) were high compared to previous studies (21.9–51.9%) [41], and most participants performed home-based TSsE quite consistently. This is likely because home-based TSsE using a tongue-strengthening self-exercise tool has benefits over TSE using a device, including the simple, straightforward methodology, cost-effectiveness, flexibility with the timing of exercise sessions, and accessibility to a wider range of participants. Older adults should continue as long as possible with TSE to prevent age-related tongue muscle weakness. TSsE that indicate high adherence rates may be useful in older adults to prevent age-related tongue muscle weakness. However, the frequency of drop-outs was higher in the exercise group than in the control group during the 8-week training program. The possibility that TSsE may be stressful for healthy older adults thus needs to be considered. On the other hand, no significant correlation was seen between adherence and effects of TSsE. This may be due to the overall high level of adherence in this study, which resulted in a ceiling effect.

The frequency of TSsE in this study was set to be every weekday (5 days/week). The frequency of TSE has varied among other studies, often 3–5 days/week [8]. We initially thought that a lower frequency of exercise would result in less stress on the patient. The frequency of exercise was set to weekdays so that patients could make training a habit, with a view to maximizing adherence. Since the effects of TSE were the same irrespective of whether the frequency of exercise was 3 or 5 days [13], matching the frequency of TSsE to the condition of the patient is appropriate.

Our study has some limitations that warrant consideration. First, the sample size was limited to both exercise and control groups. However, sample size in this study was comparable to that seen in other studies [7, 8]. Second, we did not perform a detailed assessment of swallowing function in this study because of the healthy status of the participants. Whether the TSsE could improve swallowing function thus remained unexamined. Third, no hypotrophy was seen among participants, because all participants received nutritional management. Previous studies have shown associations between nutritional status and tongue pressure [42–44]. TSsE thus may not be effective for individuals with inadequate nutrition. Fourth, we did not perform any assessment of subject frailty in this study. Previous studies have suggested a relationship between tongue strength and frailty [3]. When the subject is an older adult, frailty affecting muscle strength should be assessed, such as with a cardiovascular health study index [45, 46]. Fifth, adherence in this study was much higher than in previous studies. Our participants received day services for dwelling in the community independently for healthcare. Such individuals might therefore have had a higher level of health consciousness than usual, which could have represented a source of selection bias. Sixth, this study was completed with 8 weeks of training. This study did not clarify whether training effects were sustained after training. TSE using the appropriate device indicated that training effects were sustained after training [9, 11–14]. The presence of training effects from TSsE thus needs to be examined. Seventh, measuring and training were only performed on the anterior tongue and did not determine the effects of TSsE on the posterior tongue in this study. This was because the device for tongue pressure measurement used in this study had a bite block, so that the balloon had to be placed between the anterior tongue and anterior palate. To determine effects on the posterior part of the tongue, a tongue pressure measurement device such as the Iowa Oral Performance Instrument (IOPI) should be used.

## Conclusions

In conclusion, both MTP and ETP increased significantly in healthy older adults after 8-week TSsE using the tongue-strengthening self-exercise tool. Together, our findings suggest that older individuals may continue to perform training by themselves at home. Future studies should evaluate the effects of TSsE on swallowing function.

**Table 1:** Participant characteristics

	Exercise group	Control group	*P* value
Participants	16	11	
Age (years)	84.5 (75.3–87.8)	79.0 (72.0–82.0)	0.121
Sex (%)	Male 7 (44%)	Male 2 (18%)	0.231
	Female 9 (56%)	Female 9 (82%)	
Weight (kg)	56.0 (48.5–61.4)	52.6 (50.4–62.7)	0.904
Height (m)	1.53 (1.46–1.58)	1.51 (1.48–1.60)	1.000
BMI (kg/m^2^)	23.61 (21.89–25.28)	22.70 (20.81–26.81)	0.790
EAT-10	0 (0–1)	0 (0–1)	0.716
MNA-SF	14 (13–14)	13 (12–14)	0.080
MTP (kPa)	26.9 (20.1–33.2)	31.5 (25.1–35.6)	0.294
ETP (s)	6.7 (1.8–14.5)	5.9 (3.3–15.5)	0.645

Continuous variables are presented as median (interquartile range)

*BMI* body mass index, *EAT-10* Eating Assessment Tool-10, *MNA-SF* Mini Nutritional Assessment—Short Form, *MTP* maximum tongue pressure, *ETP* endurance of tongue pressure

**Table 2:** Effects of training on MTP and ETP

	Exercise group (*N* = 16)						Control group (*N* = 11)					
	MTP (kPa)		*P* value	ETP (s)		*P* value	MTP (kPa)		*P* value	ETP (s)		*P* value
Baseline	26.3	(8.9)		9.4	(9.7)		30.3	(7.8)		9.8	(8.7)	
4 weeks	29.7	(9.7)	0.117	14.2	(11.7)*	0.036	32.9	(7.6)	0.797	10.1	(5.0)	1.000
8 weeks	30.6	(8.1)*	0.020	17.4	(18.8)**	0.006	31.6	(6.4)	0.861	11.6	(6.8)	1.000

Continuous variables are presented as mean (SD)

*MTP* maximum tongue pressure, *ETP* endurance of tongue pressure

Significant difference versus baseline *P < 0.05, **P < 0.01

**Table 3:** Differences and rates of change in MTP and ETP

		Exercise group		Control group		*P* value
Difference between pre and post [post − pre]	MTP (kPa)	4.1	(1.0–8.2)	− 0.3	(− 1.5–5.8)	0.148
	ETP (s)	4.5	(− 1.0–13.3)	2.0	(− 2.0–3.5)	0.099
Rate of change [((post − pre)/pre) × 100]	MTP (%)	11.5	(3.6–42.4)	− 1.2	(− 6.0–23.4)	0.195
	ETP (%)	99.9	(− 15.8–174.4)	22.6	(− 17.5–70.9)	0.507

Continuous variables are presented as median (interquartile range)

*MTP* maximum tongue pressure, *ETP* endurance of tongue pressure
